# SIRT3/6: an amazing challenge and opportunity in the fight against fibrosis and aging

**DOI:** 10.1007/s00018-023-05093-z

**Published:** 2024-01-31

**Authors:** Wenxin Wei, Tian Li, Jinlong Chen, Zhen Fan, Feng Gao, Zhibiao Yu, Yihao Jiang

**Affiliations:** 1 School of Queen Mary, Nanchang University, Nanchang, 330031 China; 2 School of Basic Medicine, Fourth Military Medical University, Xi’an, 710032 China; 3 School of Chemistry and Chemical Engineering, Nangchang University, 999 Xuefu Rd, Nanchang, 330031 China; 4 The Hospital Affiliated to Shanxi University of Chinese Medicine, Xianyang, 712000 China; 5 Shanxi University of Chinese Medicine, Xianyang, 712046 China

**Keywords:** Fibrosis, Aging, SIRT3, SIRT6, Signal pathway

## Abstract

Fibrosis is a typical aging-related pathological process involving almost all organs, including the heart, kidney, liver, lung, and skin. Fibrogenesis is a highly orchestrated process defined by sequences of cellular response and molecular signals mechanisms underlying the disease. In pathophysiologic conditions associated with organ fibrosis, a variety of injurious stimuli such as metabolic disorders, epigenetic changes, and aging may induce the progression of fibrosis. Sirtuins protein is a kind of deacetylase which can regulate cell metabolism and participate in a variety of cell physiological functions. In this review, we outline our current understanding of common principles of fibrogenic mechanisms and the functional role of SIRT3/6 in aging-related fibrosis. In addition, sequences of novel protective strategies have been identified directly or indirectly according to these mechanisms. Here, we highlight the role and biological function of SIRT3/6 focus on aging fibrosis, as well as their inhibitors and activators as novel preventative or therapeutic interventions for aging-related tissue fibrosis.

**Graphical abstract:**

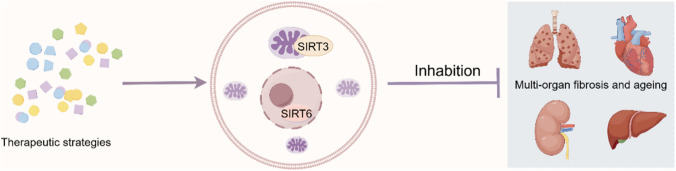

## Introduction

Fibrosis is considered to be the outcome of a chronic healing response marked by excessive deposition and formation of extracellular matrix (ECM), chronic inflammation, and loss of parenchymal cells. Activated pro-fibroblasts play a prominent role in this process [37, 55, 68, 114].

Many factors such as age, genetic factors, gender, and obesity can contribute to the development of fibrosis [30]. Fibrosis eventually affects all tissues and organs, contributing to increased morbidity and mortality from age-related diseases [111]. A complex cellular cascade induced by organ damage may underlie the coagulation disorder between ECM synthesis and ECM degradation [32]. Although the organ damage may be triggered by organ-specific ways, the fibrosis process and the involved cascades are conserved across organs [68]. The organ fibrosis is regulated by different cascades and signaling pathways including Wingless/Int (Wnt) [32] and transforming growth factor-β (TGF-β) [80, 116]. There is increasing evidence that fibrosis is the main cause of the deterioration of various human organ functions during aging.

Cell senescence refers to a permanent arrest of the cell cycle, resulting in a steady loss of the cell function to proliferate, with the continuously decreased metabolic states such as autophagy function, energy metabolism regulation and anti-stress, despite the remaining cell viability and metabolic activity [126]. Aging is accompanied by the gradually declining structural integrity of an organism [19], during which the risk of death increases [5, 22, 26, 27, 61].

Sirtuins as the niacinamide adenine dinucleotides (NAD)^+^-dependent protein deacetylases are considered the primary regulators of cell function. As a key player in regulating metabolism and preventing oxidative stress, sirtuin 3 (SIRT3) is commonly found in organs and tissues with high metabolic rate capacity, including the cardiac. Metabolic diseases and cardiac disorders related to its activity have been proposed as therapeutic targets. Maintaining genomic stability and maintaining telomere function may be accomplished by SIRT6 deacetylating histone H3 at lysine 9 and lysine 56 [99].

An important step in tissue fibrosis during chronic disease and aging is the TGF-β1-mediated fibroblasts into myofibroblasts, cells capable of synthesizing ECM. Studies have demonstrated that inhibiting pro-fibrotic TGF-β1 signaling by SIRT3 inhibits fibroblast to myofibroblast differentiation, suggesting SIRT3 is important for controlling age-related tissue fibrosis [91]. Moreover, SIRT6 functions as a key anti-aging molecule by controlling multiple cellular processes associated with aging and preventing age-induced cardiac hypertrophy and fibrosis [76]. This paper summarizes the biological structure and function of SIRT3 and SIRT6, and describes the role of SIRT3 and SIRT6 in fibrosis and aging.

## SIRT family

A sirtuin is a NAD^+^-dependent class III histone deacetylase that deacetylates histones with NAD^+^. SIRTs can exert a regulatory role in a wide range of physiological and pathological processes, including energy production, oxidative stress, mitochondrial homeostasis, cell aging and apoptosis, DNA damage, playing a prominent role in the pathogenesis progression [7, 44, 70, 77, 78].

To date, seven SIRTs subtypes, SIRT1 7, have been identified in mammalian cells [110]. SIRT1, SIRT6, and SIRT7 are located in the nucleus, SIRT2 are located in the cytoplasm, and SIRT3, SIRT4, and SIRT5 are located in the mitochondria. A translocation of SIRT1 to the cytoplasm and a relocalization of SIRT3 between mitochondria and nuclei can occur under certain conditions [85].

Different SIRTs subtypes exert different functions. SIRT1, SIRT2, SIRT3, and SIRT-7 mainly exhibit the properties of NADH-dependent deacetylases. SIRT4 and SIRT6 may function as deacetylases and ADP-ribosomal transferases. SIRT5 has weak deacetylation activity, yet it has strong desuccinylation and depropanediylation activity [106]. As mitochondrial SIRTs, SIRT3/4/5 play an important role in mitochondrial biogenesis and the regulation of oxidative stress. SIRT6, a nuclear deacetylase, plays a role in ADP-ribosyltransferase activity, inflammation and metabolism [25]. Intriguingly, SIRT6 and SIRT3 maintain each other’s levels, SIRT3 inhibits oxidative stress, and SIRT6 activates SIRT3 transcription by upregulating nuclear factor erythroid 2 (NF-E2) -related factor 2 (Nrf2) -dependent transcription [42].

### Structure and function of SIRT3

The SIRT3 gene is located on chromosome 11p15.5, a chromosome region associated with longevity [2]. Two functional domains are present on the human SIRT3 protein: a large Rossmann folding and NAD^+^ binding site, and a small helix complex and zinc binding site [38]. There is a crack between the two domains where the acetylated substrate is inserted. While being transported into the mitochondria, SIRT3 is cleaved by mitochondrial matrix processing peptidase (MPP), which results in a short, more active form 28 kDa.

Among the mitochondrial SIRTs, SIRT3 is the only one with strong NAD^+^-dependent deacetylase activity [46]. According to initial reports, SIRT3 is primarily responsible for regulating acetyl bodies in mitochondria. In the mitochondrial electron transport chain (ETC), SIRT3 directly binds to succinate dehydrogenase A and ATP synthase (Complex V) of complex I and II and regulates them, thereby vigorously raising ATP levels [60]. In energy-demanding cells, SIRT3 plays an important role in mitochondrial function and cell metabolism, such as fatty acid oxidation, tricarboxylic acid cycle (TCA), and ETC [67, 123]).

SIRT3 regulates core mitochondrial processes, but its function may differ in tissues contributing to fuel production and fuel utilization, depending on the metabolic pathways involved [18]. Thus, SIRT3 may play distinct roles at the tissue level as well as at the cellular level. In previous studies, SIRT3 deficiency caused mitochondrial respiration impairment and elevated reactive oxygen species (ROS) production in myoblasts and cancer cells [6]. SIRT3 has been speculated to be associated with human longevity, and studies have demonstrated the reduced SIRT3 expression in sedentary older adults compared to younger adults [82].

### Structure and biological function of SIRT6

The human SIRT6 gene is located at 13.3 of the short arm of chromosome 19 and consists of 8 exons, with exon 8 being the longest at 838 bases. On the other hand, exon 4 is the shortest with only 60 bases. The SIRT6 protein molecule comprises 355 amino acid residues and has a molecular weight of 39.1 kDa [63]. Structurally, SIRT6 consists of an N-terminal extension (NTE), a C-terminal extension (CTE), and a structurally conservative central domain. The NTE is associated with its catalytic activity, which is crucial for chromatin binding and the deacetylation of lysine 9 and 56 (H3K9 and H3K56) of the intrinsic histone H3. The CTE is essential for nuclear localization and recognition of nucleosome DNA. Both extensions play an important role in nucleosome binding [97]. Unlike other Sirtuin proteins, SIRT6 lacks the classical zinc finger binding sequence, Rossmann folding structure, and highly flexible NAD^+^ binding ring in its central domain. However, SIRT6 possesses a unique unfolded zinc finger binding domain and a highly stable single helix structure. Because of this unique characteristic, SIRT6 binds NAD^+^ with high affinity, even in the absence of a deacetylation substrate. This feature may explain why SIRT6 effectively promotes the ADP-ribosyltransferase reaction [24, 73].

SIRT6 exhibits both ADP-ribosyltransferase and NAD^+^-dependent histone deacetylase activities. It can utilize NAD^+^ as a substrate for intramolecular ADP-ribosylation. The currently identified glycosylation substrates of SIRT6 mainly include the K521 site of PARP1 and the nuclear helper inhibitor KAP1. As for histone deacetylation, three substrates have been identified: lysine 9, 18, and 56 of histone H3 (H3K9, H3K18, and H3K56). SIRT6 can be actively recruited to target gene promoters to inhibit the transcriptional activity of these genes through deacetylation of H3K9, H3K18, or H3K56 sites. This process helps maintain genomic stability, telomere integrity, promotes DNA repair, and prevents aging [9]. In addition, SIRT6 can deacetylate forkhead box O1 (FoxO1), associated factor histone acetyltransferase 5 (general control non-derepressible-5, GCN5), and several non-histone substrates such as C-terminal binding protein interacting proteins (CTIP). These deacetylation events further contribute to the modulation of glucose homeostasis, among other processes [66, 96].

The regulation of SIRT6 abundance is complex and precise in vivo. At the transcriptional level, SIRT6 expression is significantly activated by pharmacological inhibition of poly ADP-ribose polymerase 1 (PARP1) [108]. C-FOS and SIRT1-FOXO3a-NRF1 (SFN) complexes can also upregulate SIRT6 expression by binding to SIRT6 promoters [29]. Endogenous microRNAs (miRNAs), including miR-33a/b, miR-122, miR-330-5p, and miR-495, regulate SIRT6 translation by binding to its 3'-untranslated region (UTR) [87]. SIRT6 protein stability is largely controlled by proteasome-dependent degradation pathways.

## Fibrosis

**Fig. 1:**
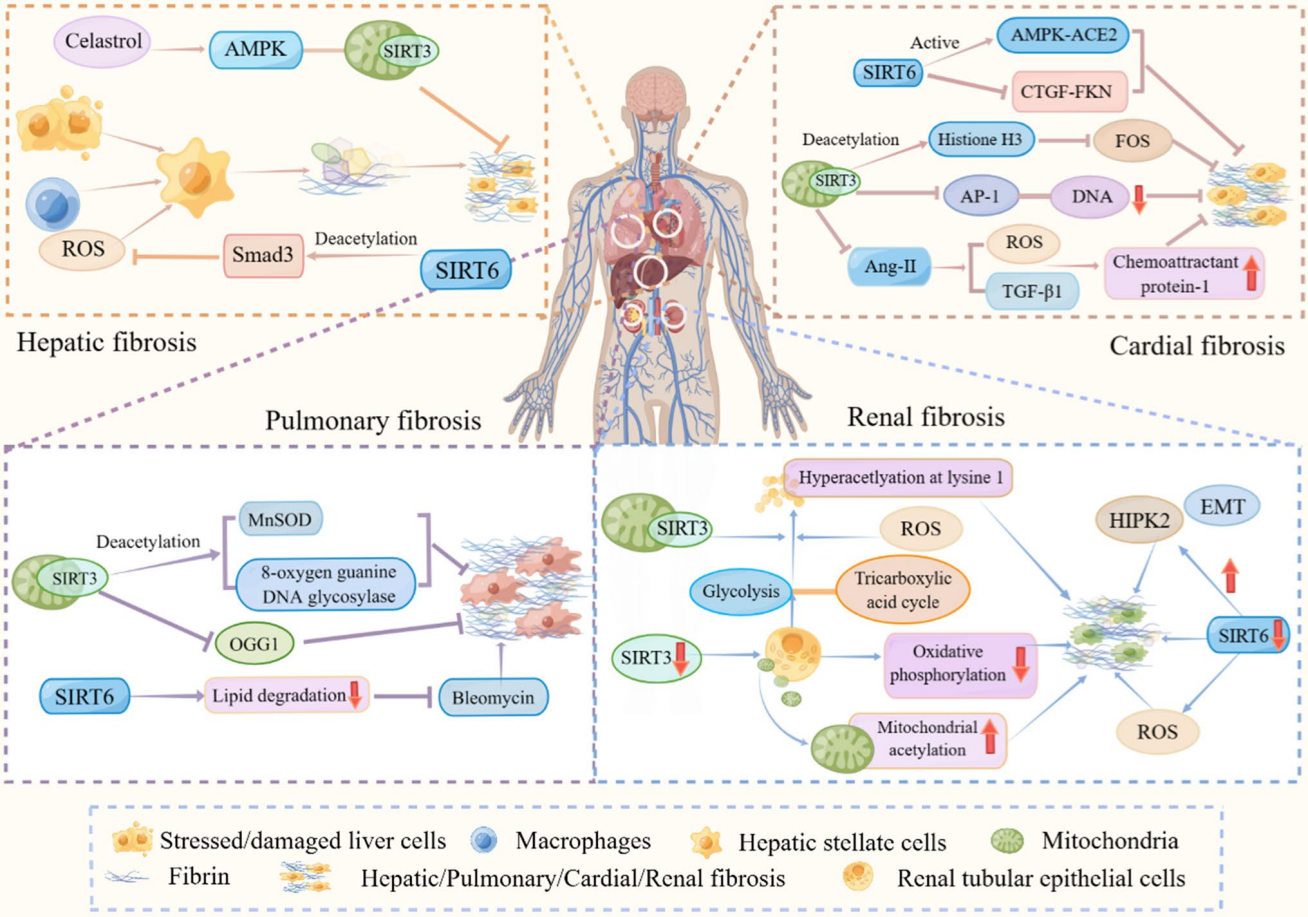
The role of SIRT3 and SIRT6 in several tissue fibrosis diseases. Mechanically, tissue fibrosis diseases include liver fibrosis, kidney fibrosis, lung fibrosis, and cardiac fibrosis

The aging process predisposes people to fibrosis and can affect many tissues (Fig. 1) [8]. SIRT3 and SIRT6 have been found to play important roles in tissue fibrosis (Table 1).

**Table 1:** A summary of the association between fibrosis diseases and SIRT3/6

Disease type	Cell	Pathway of activation		Antifibrotic drugs	References
		SIRT3	SIRT6		
Hepatic fibrosis	HSC	Activation of AMPK-SIRT3 signaling	SIRT6 inhibits TGF-β by deacetylating specific sites (K333 and K378) on Smad3	Celastrol	[107, 125]
Renal fibrosis	TECs	Regulation of glycolysis and TCA cycle and other energy sources	Down-regulate HIPK2 at the post-transcriptional level	Honokiol	[52, 123]
Pulmonary fibrosis	AEC	Deacetylating MnSOD and mitochondrial 8-oxygen guanine DNA glycosylase; Inhibits injury and apoptosis by inhibiting OGG1 acetylation at K338/341	Promote lipid degradation, thereby increasing energy supply and reducing lipid peroxide levels; Inhibits NF-κB signaling pathway and blocks TGF-β1	Bayicalein	[35, 36, 121]
Cardiac fibrosis	CF	Inhibit FOS transcription and reduce AP-1 DNA binding activity; Reducing the production of ROS and TGF-β1 by inhibiting Ang-II; Activation and regulation of ROS-NF-κB up regulates MCP-1;	Activating AMPK- ACE2 signaling and inhibiting the CTGF- CTGF pathway; Negatively regulate IGF-Akt signaling pathway; Inhibit the transcriptional activity and DNA binding activity of nuclear factor κB;	Daidzein LCZ696	[71, 75, 90, 124]

*HSC* hepatic stellate cells; *TECs* renal tubular epithelial cells; *AEC* alveolar epithelial cell; *TCA* tricarboxylic acid; *MnSOD* manganese superoxide dismutase; *HIPK2* homeodomain-interacting protein kinase 2; *CF* cardiac fibroblasts; *MCP-1* monocyte chemotactic protein-1; *AMPK* adenosine 5′-monophosphate-activated protein kinase; *ACE2* angiotensin-converting enzyme 2; *CTGF* connective tissue growth factor; *CTGF* connective tissue growth factor; *AP-1* activator protein-1

### Hepatic fibrosis

Hepatic fibrosis occurs when stressed or damaged liver cells and activated macrophages (Kupffer cells) stimulate the activation of hepatic stellate cells, which then secrete excessive ECM components, including type I and type III collagen, leading to the formation of liver fibrosis [84]. Hepatic fibrosis is a common feature of various chronic liver diseases and can progress to cirrhosis, liver failure, and hepatocellular carcinoma [16, 17].

SIRT3, which is predominantly located in the mitochondria, has been strongly associated with oxidative stress and liver-related diseases [62]. It has been shown that adenosine 5′-monophosphate-activated protein kinase (AMPK), a protein involved in the pathophysiology of liver fibrosis [41], can reduce liver fibrosis [33]. SIRT3 has been identified as a downstream effector of AMPK in several disease models, and activation of the AMPK-SIRT3 signaling pathway helps improve mitochondrial function, thereby alleviating disease progression. The anti-fibrotic effect of celastrol, which is attributed to its anti-inflammatory properties, depends on the activation of AMPK-SIRT3 signaling. Celastrol acts as an anti-fibrotic agent by suppressing inflammation, and its effects are believed to be mediated by the activation of AMPK-SIRT3 signaling. Depletion of AMPK or SIRT3 compromises the anti-inflammatory effects of celastrol [107].

Studies in mice fed a high-fat, high-fructose diet for 16 weeks and in liver samples from patients with non-alcoholic steatohepatitis (NASH) have shown that SIRT6 plays a regulatory role in the progression of NASH to liver fibrosis. Hepatocyte-specific knockout of SIRT6 in mice aggravated liver fibrosis, and reduced SIRT6 expression levels were observed in the livers of NASH patients as the disease progressed to fibrosis [39, 125]. It has been demonstrated that SIRT6 inhibits Smad3 activation, a member of the Smad family involved in TGF-β signaling, through H3K9 deacetylation, thereby suppressing the expression of key genes related to liver fibrosis and contributing to fibrosis regression [64]. Mechanistic studies have revealed that SIRT6 inhibits TGF-β-induced activation of hepatic stellate cells by deacetylating specific sites (K333 and K378) on Smad3, resulting in the downregulation of liver fibrosis-related genes and the inhibition of fibrosis progression [125].

### Renal fibrosis

An important regulator of mitochondrial function, SIRT3 participates in the injury and repair processes of acute kidney injury (AKI). It has been suggested that SIRT3 may play a significant role in the early stages of fibrosis following ischemia–reperfusion injury (IR-AKI) by regulating mitochondrial dynamics. Furthermore, deficiency of SIRT3 can potentially worsen renal insufficiency and promote renal fibrosis [13]. Proximal renal tubular epithelial cells (TECs), which are rich in mitochondria and heavily rely on mitochondrial oxidative phosphorylation as their primary energy source, are particularly affected by the dysregulation of SIRT3. In the initial stages of renal fibrosis, decreased expression of SIRT3 is accompanied by increased acetylation of mitochondria isolated from TECs. Studies using SIRT3 knockout mice have shown that these mice are more susceptible to renal fibrosis, particularly characterized by high levels of acetylated mitochondrial proteins [13]. Interestingly, the administration of honokiol has been found to activate SIRT3, leading to improved acetylation and prevention of renal fibrosis. Furthermore, in the context of unilateral ureteral obstruction, a condition associated with renal fibrosis, it has been observed that most renal proteins, accounting for 26.76% of mitochondrial proteins, undergo hyperacetylation. These hyperacetylated proteins are localized within a wide range of mitochondrial pathways. Notably, pyruvate dehydrogenase E1α (PDHE1α), a crucial link between glycolysis and the TCA cycle, undergoes hyperacetylation at lysine 1 in TECs following stimulation with TGF-β. Importantly, this process is regulated by SIRT3 [123].

In the context of AKI, studies have demonstrated that overexpression of SIRT6 can prevent its occurrence [54]. In the context of renal tubular epithelial cells, depletion of SIRT6 leads to aggravated epithelial-mesenchymal transition (EMT), accompanied by upregulation of homeodomain-interacting protein kinase 2 (HIPK2). A protein kinase called HIPK2 is involved in multiple molecular pathways that lead to cell death and development. It is noteworthy that this protein kinase can regulate a variety of pro-fibrotic pathways, such as Wnt/β-catenin, TGF-β and Notch, which are involved in kidney, lung, liver, and heart fibrosis [23]. Interestingly,, SIRT6 has the ability to down-regulate HIPK2 at the post-transcriptional level [52].

### Pulmonary fibrosis

Pulmonary fibrosis is a severe and chronic interstitial lung disease for which effective treatments are limited. Apoptosis and mitochondrial dysfunction in alveolar epithelium cells are key factors in idiopathic pulmonary fibrosis and asbestosis. Through deacetylation of manganese superoxide dismutase (MnSOD) and mitochondrial 8-oxygen guanine DNA glycosylase, SIRT3 plays a partial role in mitochondrial reactive oxygen species removal. By inhibiting acetylation of OGG1 at K338/341, SIRT3 counteracts mtDNA damage and apoptosis induced by reductive oxidant expression. Conversely, silencing of SIRT3 promotes these detrimental effects, ultimately leading to pulmonary fibrosis. Deficiency of SIRT3 contributes to increased mitochondrial DNA damage and apoptosis in alveolar epithelial cells, exacerbating the progression of pulmonary fibrosis [35]. Induction of SIRT3 expression by bayicalein reduces lung fibroblast senescence and fibrosis induced by bleomycin [36].

SIRT6, on the other hand, has demonstrated its ability to reduce fibrosis in various organs. In the context of pulmonary fibrosis, SIRT6 has been found to inhibit bleomycin-induced injury in alveolar epithelial cells both in vitro and in mice. High-throughput sequencing studies have revealed that SIRT6-overexpressing lung tissue exhibits enhanced lipid catabolism. SIRT6 mitigates bleomycin-induced ectopic lipid toxicity by promoting lipid degradation, thereby increasing energy supply and reducing lipid peroxide levels. Notably, peroxisome proliferator-activated receptor α (PPARα) has been identified as a critical mediator of SIRT6’s effects on lipid catabolism, anti-inflammatory response, and anti-fibrotic signaling. These findings suggest that targeting the SIRT6-PPARα-mediated lipid catabolic pathway holds promise as a potential therapeutic strategy for pulmonary fibrosis and related disorders [31]. In addition, other studies have demonstrated that SIRT6 inhibits NF-κB signaling pathway and blocks TGF-β1-induced lung myofibroblast differentiation [95, 124].

### Cardiac fibrosis

Cardiac fibrosis is a common patho-physiological remodeling process, which greatly affects the structure and function of the heart and further causes heart failure. Abnormal proliferation, differentiation and migration of cardiac fibroblasts are responsible for excessive deposition of ECM in cardiac muscle [57]. Acetylation plays an important role in the development of cardiac fibrosis by regulating various pathogenic conditions, including oxidative stress, mitochondrial dysfunction and energy metabolism disorders [57].

SIRT3 is expressed at high levels in the heart and improves heart health by regulating cardiac energy [67]. Studies have demonstrated that SIRT3 inhibits inflammation and fibrosis in cardiomyocytes by promoter specific deacetylation of histone H3 lysine K27 to inhibit FOS transcription and reduce activator protein-1 (AP-1) DNA binding activity [71]. Su et al. found that cardiac fibrosis was partly achieved by the mechanism of SIRT3 inducing ferroptosis in myofibroblasts through p53 acetylation [89]. Pericytes are the progenitors of myofibroblasts and fibroblasts and contribute to the deposition of ECM [21]. In another study by Su et al., SIRT3 knockdown was found to promote angiotensin II (Ang-II)-induced NADPH oxidase-derived ROS formation and increase the expression of TGF-β1. This suggests that Ang-II-induced myocardial fibrosis may involve both SIRT3-mediated transformation of pericyte into myofibroblast/fibroblasts and ROS-TGF-1 activity [90]. Cardiovascular remodeling due to obesity involves structural and functional disorders, in which cardiac inflammation and fibrosis play a critical role. Studies have found that SIRT3 upregulates monocyte chemoattractant protein-1 (MCP-1) by activating and regulating ROS-NF-κB, thereby inhibiting cardiac inflammation and fibrosis [28]. Daidzein (DAI), an isoflavone found in soy foods, has antioxidant and anti-inflammatory properties. DAI not only affects cardiac energy metabolism by regulating SIRT3 but also plays an antioxidant role through the SIRT3/FOXO3a pathway [48]. Therapeutic hypothermia intervention may inhibit inflammation and fibrosis by modulating SIRT3/NLRP3 signaling pathway [119]. Through regulation of the SIRT3/MnSOD pathway, LCZ696 ameliorates pathological cardiac remodeling caused by oxygen stress and pressure overload [75].

SIRT6 is an important regulator of cardiovascular function in health and disease. Zhang et al. demonstrated that SIRT6 negatively regulates pathological remodeling, fibrosis, and myocardial injury by activating AMPK-angiotensin-converting enzyme 2 (ACE2) signaling and inhibiting the connective tissue growth factor (CTGF)-pro-inflammatory chemokine fractalkine (FKN) pathway [124]. In addition, Sundaresan et al. found that SIRT6 knockdown can enhance histone H3 lysine 9 (H3K9) acetylation and c-Jun promoter transcriptional activity, resulting in the over-activation of a variety of IGF signaling related genes. These results indicate that SIRT6 can negatively regulate IGF-Akt signaling pathway and reduce myocardial hypertrophy and myocardial fibrosis [58]. The differentiation of cardiac fibroblasts into myofibroblasts represents a key event in cardiac fibrosis and contributes to pathological cardiac remodeling. SIRT6 deletion has been reported to induce the transcriptional activity and DNA binding activity of nuclear factor κB, further exacerbating Ang-II-induced myofibroblast differentiation [100, 112]. In addition, SIRT6 has also been shown to enhance mitochondrial biogenesis and mitophagy by deacetylation and inhibition of Sgk1, capable of ameliorating the cardiotoxicity induced by the anthracycline doxorubicin [74].

## SIRT3/6 and aging

Ageing is a natural process characterized by the gradual decline in structural integrity of an organism over time, resulting in decreased functioning and an increased risk of biological death [61]. Cellular senescence refers to a permanent state of cell cycle arrest, where cells lose their proliferative capacity while maintaining viability and metabolic activity [126]. Ageing has emerged as a major risk factor for various human diseases, including diabetes, cancer, cardiovascular disease, and neurodegenerative disorders. Cellular senescence can be categorized into two types: replicative senescence and stress-induced premature senescence (SIPS). Replicative senescence occurs due to the cessation of cell division, which is a consequence of telomere depletion [15]. On the other hand, stressors such as oxidative stress and DNA damage can induce SIPS, leading to growth arrest within a few days. It is worth noting that SIPS does not necessarily involve telomere shortening [69]. During cellular ageing, functions like autophagy, energy metabolism regulation, stress resistance, and metabolic status gradually decline.

### SIRT3 and aging

SIRT3 is one of the first genes identified to extend lifespan [40]. As early as 2003, SIRT3 was reported to be associated with longevity in humans [82]. Scientists have demonstrated that mice without SIRT3 have significantly shorter lifespans and spontaneously develop cancer, metabolic syndrome, cardiovascular disease, and neurodegenerative diseases [3]. Clinical studies have indicated that the decline in SIRT3 activity in the elderly is mainly attributed to the reduction in NAD levels, which can be partially offset by appropriate activities [14]. Furthermore, SIRT3, as a major mitochondrial deacetylase, has been found to increase the acetylation of mitochondrial proteins in various tissues of knockout mice [18].

Multiple studies have posited that the advantageous impacts of SIRT3 on the processes of aging and disease primarily occur through its facilitation of ROS clearance [86]. SIRT3 is involved in multiple antioxidant pathways. SIRT3 actively participates in numerous antioxidant pathways. The ETC serves as a significant generator of ROS, and SIRT3 can indirectly diminish ROS production by regulating the efficiency of the ETC. Furthermore, SIRT3 directly modulates the activity of various superoxide scavengers through deacetylation, thereby mitigating superoxide production and averting oxidative stress (van [103]. In addition, the transcription coactivator PGC-1α and the transcription factor FOXO3a are involved in the regulation of antioxidant enzymes expression by SIRT3 [92].

### SIRT6 and aging

SIRT6 is a nucleolar chromatin-associated protein that plays a crucial role in stabilizing the genome and telomeres, thereby preventing premature cell aging [66]. Animal studies have demonstrated that male mice with ineffective SIRT6 exhibit a phenotype of premature aging, while mice overexpressing SIRT6 show an extended lifespan [41, 66]. The deacetylation activity of SIRT6 is essential for maintaining genomic stability. As a result of SIRT6 deacetylation of H3K9ac, WRN is stabilized in telomere chromatin, preventing replication-related telomere defects, fusion of end-to-end chromosomes, and premature cell aging [66]. SIRT6 has been found to engage in interactions with the RELA subunit of NF-κB, resulting in the deacetylation of H3K9ac at the promoter region of NF-κB target genes [9]. This process effectively hinders cell senescence. In addition, SIRT6 recruits the chromatin remodeler SNF2H to the DNA cleavage site, leading to the deacetylation of histone H3K56ac. This mechanism serves to prevent genomic instability and facilitates the repair of damaged sites through chromatin remodeling [104].

In human cells, SIRT6 is indispensable for the maintenance of telomere position effect silencing and plays a crucial role in preserving the structure of silenced telomere chromatin [98]. SIRT6 promotes the deacetylation of H3K18ac, which leads to the silencing of peripheral centromeric heterochromatin and prevents abnormal accumulation of peripheral centromeric transcripts [96].

Furthermore, SIRT6 regulates glucose homeostasis and NAD metabolic balance, contributing to the slowing down of the aging process. As a result of increased lipolysis and elevated precursor levels, SIRT6 maintains the youthfulness of both the gluconeogenesis and tricarboxylic acid (TCA) cycles [81, 109].

## Pathological mechanism of SIRT3/6 in fibrosis and aging

**Fig. 2:**
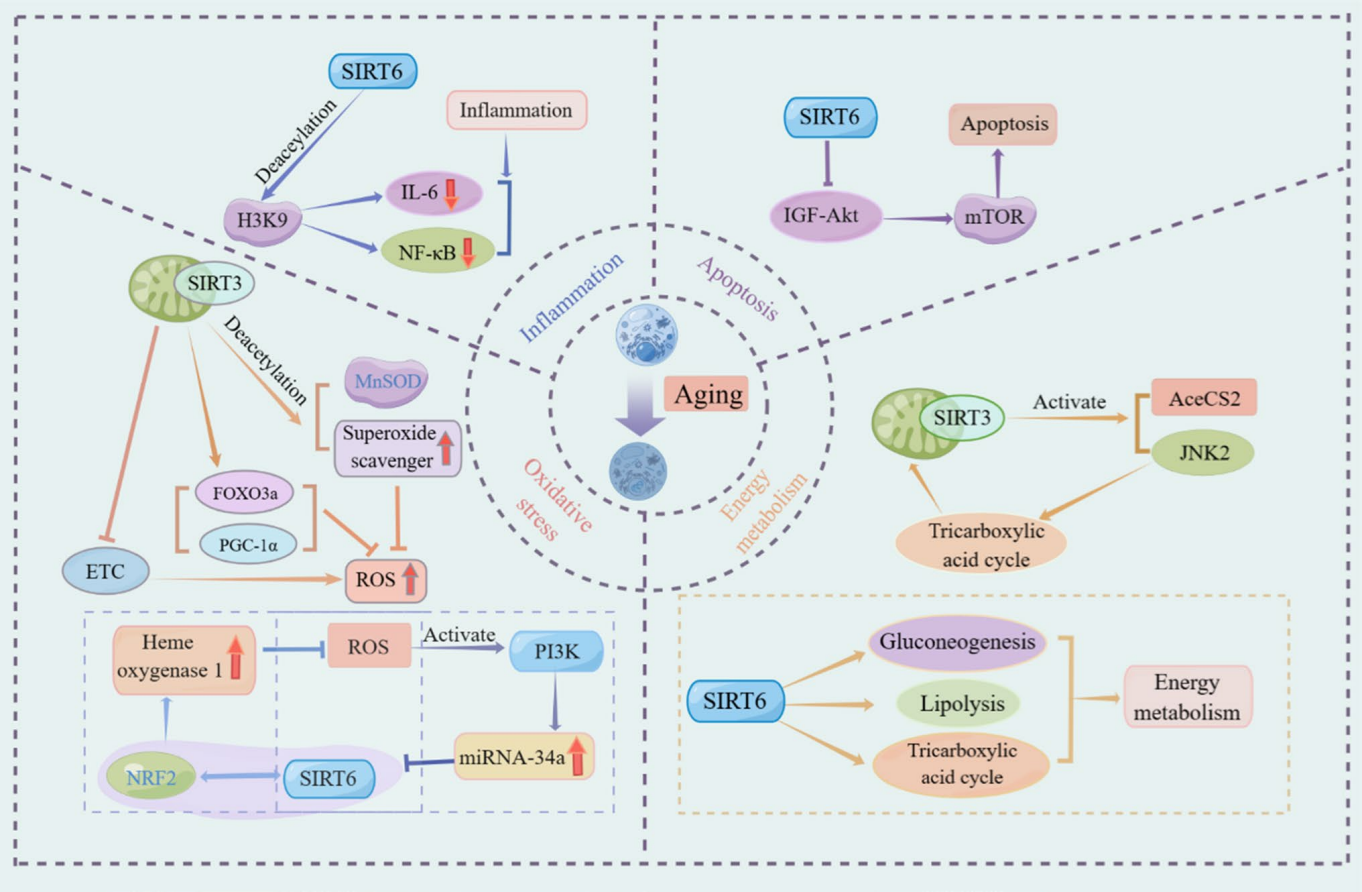
Schematic representation of the pathological mechanism of SIRT3/6 in fibrosis and aging. The pathological process of fibrosis is characterized by inflammation, oxidative stress, and apoptosis and energy metabolism. *ETC* electron transport chain; *MnSOD* manganese superoxide dismutase; *PI3K* phosphatidylinositol 3-kinase

The pathological process of fibrosis is characterized by inflammation, oxidative stress, and apoptosis and energy metabolism (Fig. 2).

Fibrosis is characterized by the aberrant accumulation of ECM proteins within the interstitial space, representing a fundamental pathological reaction to persistent inflammation. SIRT6 was recruited to the promoter regions of NF-κB target genes, leading to the suppression of gene expression. This inhibition occurs through the deacetylation of histone H3K9 at the target gene’s promoter by SIRT6 [43]. Sirt6 knockout mice exhibited obesity-related insulin resistance and increased inflammation in adipose tissue [45]. The loss of SIRT6 in macrophages resulted in the activation of NF-κB, leading to the production of IL-6 in the endothelium. This, in turn, activated the positive feedback loop involving STAT3 and NF-κB [47]. The interaction between SIRT6 and NF-κB activation enhances the pro-inflammatory M1 polarization of macrophages in the bone marrow and augments the migratory capacity of macrophages toward adipose tissue.

SIRT3 plays a critical role in mitigating mitochondrial oxidative stress through direct regulation of MnSOD [79, 94]. Furthermore, SIRT3 and SIRT4 collaborate to maintain mitochondrial NAD levels and safeguard against cell death following induced stress [117]. SIRT6 has been identified as a crucial element in the process of aging and age-related illnesses. SIRT6 interacts with NRF2, a transcription factor that regulates the expression of antioxidant genes, including heme oxygenase 1 in human mesenchymal stem cells [72]. Therefore, SIRT6-mediated activation of NRF2 protects cells from decay by protecting them from oxidative stress. Phosphatidylinositol 3-kinase (PI3K), as one of the central regulators of aging, plays a key role in the regulation of aging-related diseases [59]. Through PI3K activation, miR-34a becomes upregulated in epithelial cells in response to oxidative stress. Oxidative stress triggers the activation of miR-34a, leading to a decrease in the expression of SIRT6 and its abilities to combat aging. During periods of hyperosmotic stress, the cell utilizes aldose reductase (AR) to control its stress reactions [101, 102]. It is interesting to note that the increased AR expression under hyperosmotic stress is the result of SIRT6-mediated regulation. By overexpressing SIRT6, serum levels of IGF1 were reduced, IGF-binding protein 1 was increased, and major components of the IGF1 signaling pathway were phosphorylated [41].

Through autophagy, damaged cells are eliminated from the body, preventing cellular senescence [83]. IGF-Akt signaling through mTOR exerts a negative regulation on the process. It is reported that SIRT6 triggers autophagy in human bronchial epithelial cells by attenuating IGF-Akt-mTOR signaling [93]. Induction of autophagy prevents cellular damage and the aging process, further supporting SIRT6’s role in aging.

SIRT3, known as longevity promoting sirtuin, is also known as the “guardian of mitochondria” [65]. SIRT3 regulates mitochondrial DNA integrity, mitochondrial structural dynamics, and functional homeostasis by affecting metabolism. SIRT3 exhibits deacetylase activity against hundreds of mitochondrial proteins and is able to regulate stress pathways and energy homeostasis [1]. The energetic regulation of SIRT3 is further enhanced by its role in the TCA, as it activates the functions of AceCS2 and JNK2 [12].

## Therapeutic strategies for SIRT3/6 in aging and organ fibrosis

SIRT3/6 has been shown by more and more studies to alleviate the progression of multi-organ fibrosis and is a promising target (Table 2).

**Table 2:** Therapeutic strategies for SIRT3/6 in aging and organ fibrosis

Sirtuin	Therapeutic strategies	Disease type	Pathway	References
SIRT3	Probucol	Pulmonary fibrosis	Restoring SIRT3 expression	[118]
	Baicalein	Lung fibroblast senescence and pulmonary fibrosis	Restoring SIRT3 expression and inhibiting TGF-β1/Smad signaling pathway	[36]
	Poricoic acid A	Renal interstitial fibrosis	Upregulating SIRT3 and inducing β-catenin K49 deacetylation	[11]
	Uncoupling protein 1	Renal interstitial fibrosis	Inhibit the occurrence of oxidative stress by stabilizing SIRT3; Reducing EMT and ECM accumulation	[115]
	PSG	Liver fibrosis	Regulating SIRT3-mediated NF-κB P65 expression	[10]
	Hesperetin derivative	Hepatitis and liver fibrosis	Activating AMPK/SIRT3 pathway	[49]
	γ-man	Liver fibrosis	Induce SIRT3 to inhibit NAD(P)H oxidase activity; Enhanced SIRT3 expression and decreased HMGB1 expression; Reduced accumulation of type I collagen and α-SMA	[105]
	Therapeutic hypothermia	Myocardial inflammation and fibrosis	SIRT3/NLRP3 signaling pathway	[119]
	Hydrogen sulfide	Myocardial fibrosis	Enhanced the activity of the SIRT3 promoter and increased SIRT3 mRNA expression;	[56]
SIRT6	Yishen Tongluo formula	Renal fibrosis	Regulating SIRT6/TGF-β1/Smad2/3 signaling pathway, promoting TGF-β1 degradation; Inhibiting the expression of type I collagen, α-smooth muscle actin, type IV collagen and fibronectin	[122]
	Calorie restriction	Age-dependent renal degeneration and replicative senescence of human fibroblast	Enhancing SIRT6 expression; SIRT6 interacts with NF-κB to regulate inflammation and apoptosis	[120]
	Hydrogen sulfide	Cardiomyocyte senescence and diabetic myocardial fibrosis	Activate cystathionine-lyase and autophagy through SIRT6/AMPK signaling pathway;	[53]
	OSS-12816 (SIRT6-specific inhibitor)	Myocardial fibrosis	Increase the levels of inflammatory factors and ROS; aggravate the apoptosis and fibrosis	[34]

Zhang et al. found that probucol, as a cholesterol-lowering drug with strong antioxidant properties, improved EMT and pulmonary fibrosis by restoring SIRT3 expression [118].In addition, a recent study found that baicalein attenuates bleomycin-induced lung fibroblast senescence and pulmonary fibrosis by restoring SIRT3 expression and inhibiting TGF-β1/Smad signaling pathway [36]. Renal interstitial fibrosis is a common pathway for the progressive development of chronic renal diseases (CKD) with different etiology, and is the main pathological basis leading to end-stage renal disease. Poricoic acid A is an anti-fibrotic drug isolated from *Poria cocos*. It was shown to attenuate renal fibroblast activation and interstitial fibrosis by upregulating SIRT3 and inducing β-catenin K49 deacetylation [11]. Uncoupling protein 1 is a nuclear encoded protein located in the inner mitochondrial membrane, which has been shown to inhibit the occurrence of oxidative stress by stabilizing SIRT3, thereby reducing EMT and ECM accumulation, and ultimately alleviating renal interstitial fibrosis [115]. Liver fibrosis, a chronic inflammatory healing reaction, progresses to hepatocirrhosis without effective intervention. Physion 8-*O*-β-glucopyranoside (PSG), an anthraquinone derived from *Rumex japonicus* Houtt, was shown to be able to increase the enzymatic activity and promoter activity of SIRT3 in fibrotic liver and activated hematopoietic stem cells. In addition, PSG significantly increased SIRT3 mRNA and protein expression. In brief, PSG can reduce inflammatory response by regulating SIRT3-mediated NF-κB P65 expression in liver fibrosis, which is an effective anti-fibrotic effect [10]. Hesperetin derivative (HD-16), a monomer compound extracted from hesperetin, was proved by Li et al. to reduce ccl4-induced hepatitis and liver fibrosis by activating AMPK/SIRT3 pathway [49]. Studies have found that γ-man is a strong candidate for the treatment of oxidative stress-induced liver fibrosis. γ-man can induce SIRT3 to inhibit NAD(P)H oxidase activity, thereby reducing oxidative stress in cells. In addition, γ-man enhanced SIRT3 expression and decreased HMGB1 expression, resulting in reduced accumulation of type I collagen and α-SMA in the liver [105]. Zhang and colleagues demonstrated that therapeutic hypothermia inhibits inflammation and fibrosis through SIRT3/NLRP3 signaling pathway to protect myocardial ischemia—reperfusion injury in an isolated rat heart model [119]. Liu et al. explored the role of hydrogen sulfide through the detection of SIRT3 myocardial fibrosis. They found that NaHS enhanced the activity of the SIRT3 promoter and increased SIRT3 mRNA expression. Altogether, NaHS attenuated myocardial fibrosis through oxidative stress inhibition via a SIRT3-dependent manner [56].

SIRT6 regulates DNA repair, glucose and lipid metabolism, cellular senescence, and inflammation. Studies have found that Yishen Tongluo formula significantly improves renal fibrosis by regulating SIRT6/TGF-β1/Smad2/3 signaling pathway, promoting TGF-β1 degradation, and then inhibiting the expression of type I collagen, α-smooth muscle actin, type IV collagen and fibronectin [122]. It has been found that calorie restriction can delay age-dependent renal degeneration and replicative senescence of human fibroblast WI38 by enhancing SIRT6 expression. In addition to this, SIRT6 interacts with NF-κB to regulate inflammation and apoptosis [120]. Li and colleagues demonstrated that exogenous H_2_S could activate cystathionine-lyase and autophagy through SIRT6/AMPK signaling pathway, inhibit cardiomyocyte senescence and improve diabetic myocardial fibrosis [53]. In addition, SIRT6 is also a potentially favorable therapeutic target for diabetic cardiomyopathy. SIRT6-specific inhibitor OSS-12816 can increase the levels of inflammatory factors and ROS in vitro and in vivo, and aggravate the apoptosis and fibrosis of cardiomyocytes induced by diabetes in mice [34].

## Perspective

Interstitial fibrosis is a prevalent pathological characteristic observed in various tissues during the process of aging, resulting in the gradual decline of organ functionality. Among the organs commonly affected by age-related ailments, the kidneys are particularly susceptible, rendering older individuals more prone to chronic kidney disease.

Mammalian sirtuins have emerged as a group of metabolic regulators that facilitate the connection between protein acetylation and energy metabolism. While the comprehension of the roles of distinct sirtuins across different organ levels is still in its nascent phase, there has been some advancement in identifying sirtuin targets that have a wide range of effects on cellular protection and regeneration mechanisms. Consequently, a logical progression from these discoveries is the exploration for compounds that activate sirtuins [20, 88]. Resveratrol has the ability to function as an allosteric modulator, inducing structural alterations in the substrate, consequently enhancing its affinity for sirtuins [4]. Encouraging results have been achieved in the areas of diabetes, cardiovascular disease, and neuropathy. Regarding SIRT3, magnolol has demonstrated the ability to selectively activate SIRT3, thereby exhibiting anti-inflammatory and antioxidant effects in both chronic and acute kidney disease models [50, 51].

Ageing is a major risk factor for chronic diseases and is highly associated with cardiovascular disease, cancer, metabolic disorders, and decline in organ function over time [113]. It is important to note that most fibrotic diseases become more common with age. In an increasingly aging population, the use of effective anti-fibrosis treatments is essential to extend healthy life. In recent years, the important physiological functions of SIRTs in the pathophysiology of organ fibrosis have attracted extensive attention. SIRT regulates a variety of biological functions in different processes. In addition, SIRT is considered a therapeutic target for age-related diseases. As two different subtypes of SIRTs, SIRT3 and SIRT6 play an irreplaceable role in the prevention and treatment of aging and fibrosis. Based on the description of aging and fibrosis, the function of SIRT3/6 in aging and organ fibrosis was summarized, and some prevention and treatment strategies were provided for aging and organ fibrosis.

This review also has some limitations, fibrosis and aging are complex processes and mutual influence. Insights into the regulatory mechanisms of SIRT have been extrapolated from in vitro studies, lacking more data from clinical trials. In addition, other members of the SIRT family also play an important role in fibrosis and aging, which requires further study and comparison of the interaction and interaction between SIRT family members.

The advancements made in the targeting of SIRT3/6 have shown promising potential for the development of novel therapies aimed at addressing tissue injury and fibrosis associated with aging. In addition, these investigations have elucidated the underlying mechanisms of SIRT3/6-mediated signaling pathways involved in oxidative stress, inflammation, and apoptosis across various tissues. In conclusion, the findings from previous research serve as a valuable foundation for future studies focused on the prevention and mitigation of age-related tissue fibrosis.

## Data Availability

N/A.
